# Accuracy Evaluation of Dose Warping Using Deformable Image Registration in Carbon Ion Therapy

**DOI:** 10.1016/j.ijpt.2024.100639

**Published:** 2024-12-17

**Authors:** Yuya Miyasaka, Hikaru Souda, Hongbo Chai, Miyu Ishizawa, Hiraku Sato, Takeo Iwai

**Affiliations:** 1 Department of Heavy Particle Medical Science, Yamagata University Graduate School of Medical Science*,* Yamagata, Japan; 2 Department of Radiology, Faculty of Medicine, Yamagata University*,* Yamagata, Japan

**Keywords:** Carbon-ion therapy, Deformable image registration, Dose warping, Adaptive Particle Therapy

## Abstract

**Purpose:**

The study's purpose was to use a simple geometry phantom to validate the deformable image registration (DIR) accuracy and dose warping accuracy in carbon ion radiotherapy (CIRT) and to provide an index for dosimetry in CIRT.

**Materials and methods:**

We used geometric and anatomical phantoms provided by AAPM TG-132. The DIRs of 3 different settings were performed between reference and translational images for each phantom. CIRT and photon therapy (3-dimensional conformal radiotherapy and volumetric modulated radiotherapy) treatment plans were transformed by the use of deformation vector fields calculated from the DIR of each setting. The dose distribution calculated on the basis of rigid registration between images was used as the ground truth and compared with the warped dose determined by DIR to evaluate the error.

**Results:**

The photon therapy treatment plans showed a dose warping error of <2% for a DIR error of <2 mm, whereas CIRT showed a dose warping error >10% for the same DIR accuracy. From this, even with similar DIR accuracy, the errors tended to be larger for CIRT than for photon therapy dose distributions. Due to the steepness of the CIRT dose gradient, the dose difference increased by about 5% for a DIR error of 5 mm, which was larger than the 3% dose difference generated for a 5 mm DIR error in photon therapy.

**Conclusion:**

We evaluated the relationship between DIR accuracy and dose warping accuracy in the CIRT dose distribution. Due to the steepness of the dose gradient in CIRT, we concluded that dose warping based on DIR accuracy should be required to be sufficiently higher than that in photon therapy.

## Introduction

Particle therapy has been used to treat cancer in a variety of sites with demonstrated effectiveness.^1^, ^2^, ^3^, ^4^, ^5^, ^6^ Particle therapy is characterized by the particle range and Bragg peak and enables highly concentrated dose delivery to tumors, resulting in improved tumor control and reduced toxicity.^7^, ^8^, ^9^, ^10^ In contrast to these significant benefits versus photon therapy, there are weaknesses in particle therapy. One weakness is the low robustness of the treatment plan. Houweling et al^11^ evaluated the dose changes introduced by the interfractional error of photon therapy and carbon ion radiotherapy (CIRT). Their results showed that the dose change in photon therapy was <0.5% for both the target and organ at risk (OAR), whereas CIRT showed a 10% decrease in the target dose, revealing the low robustness of particle therapy. In addition, various studies have shown low robustness in particle therapy.^12^

Adaptive particle therapy is a way to increase the reproducibility of treatment plans. There are various reports on the importance of providing adaptive particle therapy.^13^, ^14^, ^15^ Simone et al^14^ found that adaptive proton therapy can significantly reduce the OAR dose and may provide safe treatment. In photon therapy, for example, adaptive radiotherapy using a magnetic resonance image linear accelerator is already in clinical use, and good treatment outcomes have been reported.^16^, ^17^, ^18^ Adaptive particle therapy has lagged behind photon therapy in popularity, but various research and development efforts are underway, and it is believed that widespread use will soon be possible.

Among the various technical issues involved in adaptive particle therapy, this study focused on dose assessment. If the treatment plan is redesigned for each treatment fraction in adaptive particle therapy, the final dose assessment must take into account the organ movement of each treatment fraction. Deformable image registration (DIR) can achieve this. The use of DIR in photon therapy has been reported, and the evidence is becoming more established.^19^, ^20^, ^21^, ^22^ In contrast, there is a lack of evidence on the relationship between DIR accuracy in dosimetry of particle therapy, especially for CIRT. Kumagai et al^23^ reported on the use of DIR to evaluate the accumulative dose in lung cancer treatment. Kubota et al^24^ evaluated the relationship between DIR accuracy and dosimetry accuracy in pancreatic cancer. Although DIR dose assessment has been performed for each site, a quantitative evaluation of DIR accuracy and warped dose that uses basic structures and phantoms that should be used as standards for CIRT has not been reported, and it is not clear if the required accuracy is comparable to that of photon therapy. The study's purpose was to verify the relationship between DIR accuracy and dose warping accuracy for dose assessment using DIR in CIRT and to provide an index that can validate dosimetry.

## Materials and methods

### Phantom computed tomography image

**Figure 1:**
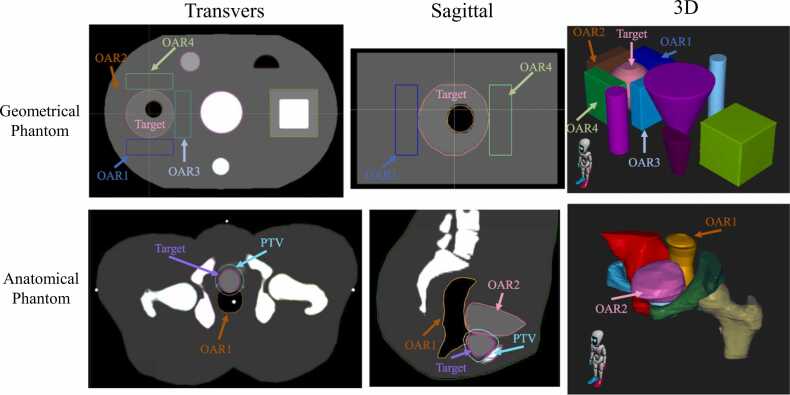
Phantom images used for evaluation and ROIs created on the images. Abbreviations: 3D, three dimensional; OAR, organ at risk; and ROI, region of interest.

**Figure 2:**
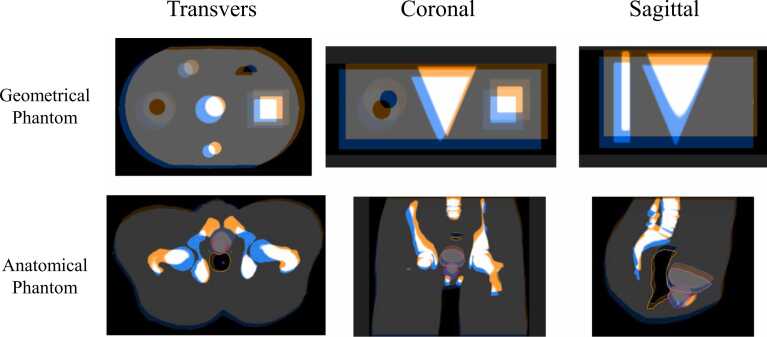
The initial misalignment of the reference image and the translational image before registration. The blue image is the reference image, and the orange image is the translation image.

In this study, the DIR evaluation phantom designated by AAPM TG-132 was used.^25^ Reference and translation images of each phantom, classified as geometric and anatomical phantoms, were used. The phantom computed tomography (CT) image and the region of interest (ROI) are shown in Figure 1. For the geometric phantom, ROIs were created for each structure, of which the spherical structure in the phantom’s right direction was designated as the target. In addition, rectangular ROIs were created in each of the 4 directions around the target, which were designated as OAR1, 2, 3, and 4. For the anatomical phantom, ROIs were created with the target in the center of the phantom and the structures located on its anterior and posterior sides designated as OAR1 and 2, respectively. ROIs were also created for high CT value areas around the target and OAR. In addition, a 5-mm margin in all directions was added to the target, which was used as the planning target volume (PTV). Figure 2 shows the initial misalignment of the reference image and the translational image before registration. In the translational image, the same phantom as in the reference image was moved in parallel, and the displacements were left-right: 0.3 cm, 1.0 cm; inferior-superior: 1.2 cm, 1.5 cm; and posterior-anterior: 0.5 cm, 0.5 cm, for the geometrical and anatomical phantoms, respectively.

### Deformable image registration description and deformable image registration accuracy evaluation

The DIR algorithm used was ANACONDA, which is included in RayStation.^26^ ANAtomically CONstrained Deformation Algorithm (ANACONDA) is an algorithm that uses both image intensity and contour information for registration. The user can select which contour information is used. In this study, DIR was performed in 3 settings for each piece of contour information used. The first is a setting using all structures defined within the body contour (DIR setting 1), the second is a setting using the target and surrounding structures or the target and OAR near the target (DIR Setting 2), and the third is a setting using the target only (DIR setting 3). The DIR for each setting was performed with the reference image as the registration reference image and the translational image as the registration moving image. The deformation grid size was set to 2.5 mm, and a correlation coefficient was used for the similarity metric. These parameters were adopted in this study because they are the default settings in the RayStation DIR settings and have been widely used in previous studies.^27^, ^28^, ^29^ The Housdorff distance (HD), mean distance to agreement (MDA), and dice similarity coefficient (DSC) of the target and OAR in each DIR setting were measured to evaluate DIR accuracy. In HD, the maximum value was evaluated. To evaluate the validity of the DIR, we compared the deformation grid provided in AAPM TG-132 with the deformation grid for each setting in this study.

### Dose calculation and dose deformation

RayStation 10A (RaySearch Laboratories AB, Stockholm, Sweden) was used to create the treatment plan. Four treatment plans were created for each of the geometric and anatomical translational phantoms. The first and second treatment plans were CIRT treatment plans with gantry angles of 0° and 90°. For the anatomical phantom, a treatment plan with PTV as the irradiation volume (PTV-based plan) and a robustly optimized treatment plan with the target as the irradiation volume with a setup uncertainty of 5 mm in all directions were created (robust plan), respectively. Robust optimization was performed as a method based on the mini-max method.^30^ The third treatment plan was a three-dimensional conformal radiotherapy (3DCRT) with 6 fields of 0°, 60°, 120°, 180°, 240°, and 300° (geometric phantom) and 6 fields of 45°, 105°, 130°, 230°, 255°, and 315° (anatomical phantom). The fourth treatment plan was single full-arc volumetric modulated arc therapy (VMAT). All treatment plans delivered 10 Gy in 1 fraction to the target, and the prescribing method was a mean dose prescription for the target or PTV. The dose calculation algorithm was pencil beam for CIRT plans and collapsed-cone convolution for 3DCRT and VMAT plans. The irradiation method for CIRT was the spot-scanning method of the full-energy scan method.^31^ The modified microdosimetric kinetic model was used as the biological dose calculation model for CIRT treatment planning.^32^, ^33^ In the CIRT plan, a beam model of a commercial carbon beam therapy system CI −1000 (Toshiba Ltd, Kawasaki, Japan) was used.^34^ This system uses a raster scanning that manages the number of irradiated particles per spot and irradiates the beam between spots without stopping.^35^ The minimum and maximum energy of the available carbon beam was 55.6 and 430 MeV.^31^ The linear accelerator for the photon therapy treatment plan was a Versa HD, and 6 MV X-rays were used. The dose distribution of each treatment plan was deformed on the basis of the deformation vector fields calculated by the DIR of each setting, and this deformed dose was warped to the reference image.

### Dose evaluation

The reference image and moving image were rigidly registered to match, and the beam parameters were fixed, with the isocenter shifted to the reference image, and the dose was recalculated as the ground truth dose distribution. We then compared the ground truth dose and the warped dose for each DIR setting on the reference image. The difference between the ground truth and warped dose of the target or PTV (D_90%_, D_95%_, D_98%_, D_2%_) and OAR (D_mean_, D_1%_, D_2%_, D_5%_, D_10%_, D_20%_, D_30%_) dose-volume histogram (DVH) parameters were calculated according to Equation (1):

(1) $$\mathrm{Difference}\left(\%\right)=\frac{{\mathrm{DVH}}_{G}-{\mathrm{DVH}}_{W}}{10}\;\times \;100\;\left(\%\right)$$

where DVH_G_ denotes the value of DVH parameter for the ground truth, and DVH_W_ denotes the value of the DVH parameter for the warped dose. The value of 10 is in the denominator because the prescription dose is 10 Gy, and the error rate was calculated for the prescription dose.

In addition, a gamma analysis was performed on the ground truth dose distribution and the warped dose for each DIR setting. Gamma analysis was performed based on global max, with criteria 3%/3 mm, 2%/2 mm, and 1%/1 mm, respectively, and thresholds 10% or greater were considered for evaluation. 3D slicer ver.5.6.2 was used as the software for gamma analysis.

## Results

**Figure 3:**
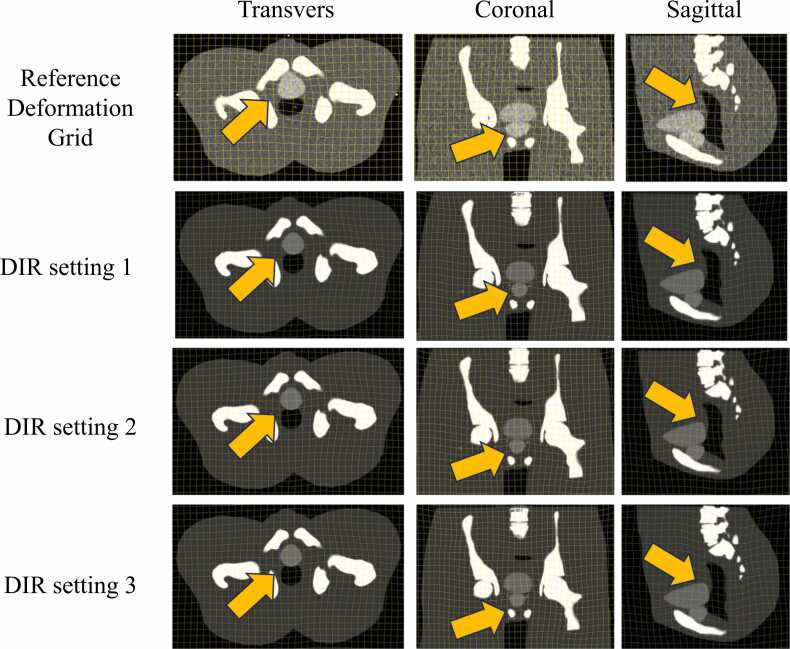
Comparison of the DIR deformation grid in this study with the deformation grid provided in TG-132. Abbreviations: DIR, deformable image registration; TG, task group.

A comparison of the deformation grid provided by TG-132 and the DIR deformation grid in this study is shown in Figure 3. The deformed areas and the amount of deformation were generally similar. Larger local deformations were identified for the deformation gird performed in this study.

**Figure 4:**
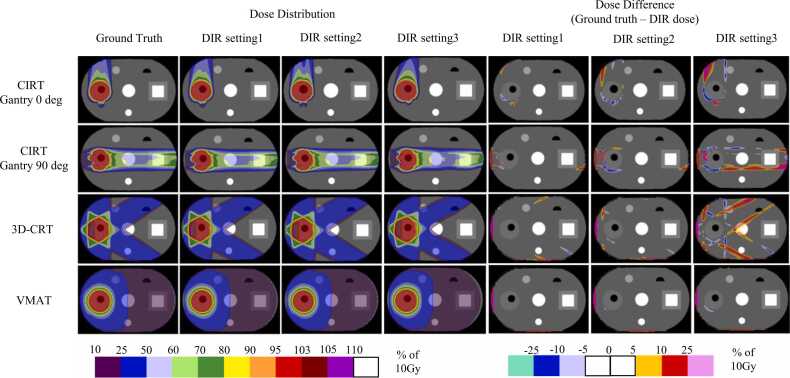
Dose distribution of ground truth, dose distribution of warped dose for each DIR setting, and map of the difference between them in the geometrical phantom. Abbreviations: 3D-CRT, three-dimensional conformal radiotherapy; CIRT, carbon ion radiotherapy; DIR, deformable image registration; and VMAT, volumetric modulated radiotherapy.

**Figure 5:**
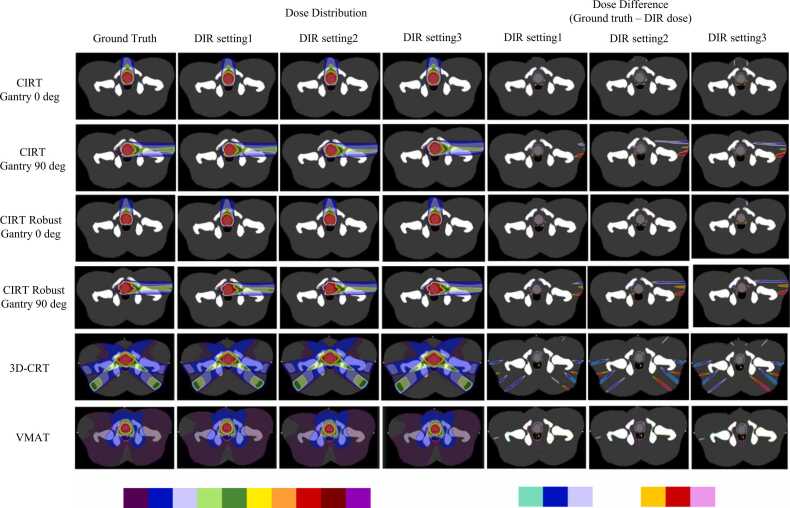
Dose distribution of ground truth, dose distribution of warped dose for each DIR setting, and map of the difference between them in the anatomical phantom. Abbreviations: 3D-CRT, three-dimensional conformal radiotherapy; CIRT, carbon ion radiotherapy; DIR, deformable image registration; and VMAT, volumetric modulated radiotherapy.

**Figure 6:**
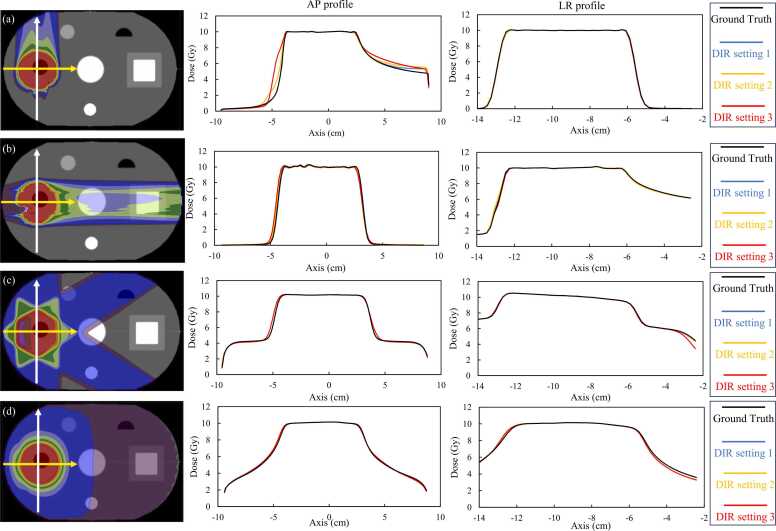
Dose distribution profiles of ground truth and warped dose at target center in geometrical phantom. (a) CIRT 0°, (b) CIRT 90°, (c) 3D-CRT, and (d) VMAT dose distribution and dose profile. Abbreviations: 3D-CRT, three-dimensional conformal radiotherapy; AP, anterior-posterior; CIRT, carbon ion radiotherapy; DIR, deformable image registration; LR, left-right; and VMAT, volumetric modulated radiotherapy.

Figure 4, Figure 5 show the dose distribution of the ground truth, the dose distribution for each DIR setting, and the difference map of each dose distribution for the geometric and anatomical phantoms, respectively. For all CIRT and photon treatment plans in any phantom, the lowest error was at DIR setting 1, and the highest error was at DIR setting 3. The errors were particularly large in the penumbral region where the dose gradient is large, but in CIRT, the errors were large not only in the penumbra but also at the end of the particle range region. The errors were fewer in VMAT than in 3DCRT or CIRT. The robust plan in CIRT for anatomical phantom shown in Figure 5 shows a similar trend compared to PTV-based plant. Figure 6 shows the dose profiles in the geometric phantom in the anterior-posterior and left-right directions centered on the target for the ground truth and warped doses for each DIR setting. CIRT showed errors in the penumbral region and on the proximal and distal sides of the spread-out Bragg peak. In 3DCRT, errors occurred in the penumbral region. Errors tended to be smaller in VMAT than in the other treatment plans. In the photon treatment plan, the error in DIR setting 3 is clearly identified, whereas in the CIRT treatment plan, the error is identified not only in DIR setting 3 but also in DIR setting 2. Table 1, Table 2 show the DVH parameter errors for the ground truth and warped dose and DIR accuracy index values, for each ROI in the geometric and anatomical phantoms, respectively. For OAR, the DIR accuracy index values for DIR setting 1 were generally the best, with DIR setting 3 tending to be the worst. In OAR, the better the DIR accuracy, the smaller the DVH parameter error, and the worse the DIR accuracy index, the larger the dose error. Compared with the parameters in the photon therapy plans, some DVH parameters in CIRT had an error ≥10%, confirming a tendency toward larger dose errors even with similar DIR accuracy. For the target, DIR accuracy was best at DIR setting 3 for both the CIRT and photon therapy plans. Target DVH parameters are less variable than OAR, depending on DIR accuracy. However, the DVH parameter errors at each DIR setting were significantly different in the D_95%_ of the 90° treatment plans in CIRT. A comparison of the anatomical phantom CIRT PTV-based plan and robust plan in Table 2 showed generally similar trends. However, there were large differences in some parameters. For example, the D_5%_ for OAR1 in the PTV-based plan was −0.41% in DIR setting 1 compared to −2.31% in the robust plan. Furthermore, for the target DVH parameters, the dose difference tended to be slightly larger for the robust plan than for the PTV-based plan.

**Table 1:** Difference between ground truth and warped dose of DVH parameters and DIR accuracy index values for each ROI in geometrical phantom.

*Carbon*		Dose difference (%)														DIR accuracy		
		Dmean		D_1%_		D_2%_		D_5%_		D_10%_		D_20%_		D_30%_		HD (mm)	MDA (mm)	DSC
ROI	DIR setting	0°	90°	0°	90°	0°	90°	0°	90°	0°	90°	0°	90°	0°	90°			
OAR1	Setting1	−0.30	−0.24	0.52	−3.94	−0.37	1.63	−1.46	−0.47	−2.84	−2.57	−0.30	−0.61	−0.07	−0.20	3.00	0.03	1.00
	Setting2	−1.60	−2.54	−0.78	−7.74	−5.57	−5.57	−8.76	−8.87	−8.54	−12.27	−1.90	−6.11	−0.57	−2.70	9.08	1.36	0.89
	Setting3	−2.60	−2.44	−4.58	−8.24	−4.77	−6.77	−12.36	−8.87	−13.34	−10.37	−4.00	−5.81	−1.07	−2.70	17.10	2.94	0.79
OAR2	Setting1	−0.45	−0.45	−2.96	0.13	−0.65	3.57	−1.69	3.91	−2.48	−0.59	−1.66	−3.85	−0.64	−0.80	0.52	0.08	1.00
	Setting2	−0.95	−1.15	1.44	3.13	−1.85	1.97	−3.89	1.51	−3.28	−3.49	−3.46	−4.65	−1.64	−1.30	9.84	1.43	0.89
	Setting3	−1.25	−1.65	−1.26	1.43	−3.55	1.47	−6.29	−0.99	−4.78	−5.39	−4.16	−5.85	−1.94	−1.60	17.78	3.26	0.78
OAR3	Setting1	−0.08	0.03	1.39	0.17	0.76	0.12	−0.17	0.11	−0.54	0.03	−0.19	−0.07	−0.04	−0.01	0.33	0.02	1.00
	Setting2	0.22	−0.17	3.79	0.17	3.36	0.22	2.53	0.21	0.66	0.03	0.01	0.03	−0.04	0.09	3.00	0.23	0.98
	Setting3	−1.08	−0.27	2.49	−0.03	1.26	−0.08	−1.87	−0.29	−5.04	−0.37	−3.39	−0.27	−1.64	−0.41	10.98	2.54	0.80
OAR4	Setting1	0.16	−0.13	0.09	2.46	−0.07	1.06	0.04	1.24	−0.04	1.18	−0.02	−0.71	0.05	−0.51	3.00	0.04	1.00
	Setting2	0.16	−0.03	1.79	4.76	1.13	4.46	0.64	4.64	0.26	2.88	−0.12	−1.41	−0.25	−1.71	9.66	1.16	0.91
	Setting3	−1.14	−3.03	1.09	3.46	0.53	2.46	−0.36	−1.16	−1.04	−5.12	−1.62	−9.31	−1.95	−7.21	16.90	3.46	0.74
														
		D_90%_		D_95%_		D_98%_		D_2%_								HD (mm)	MDA (mm)	DSC
		0°	90°	0°	90°	0°	90°	0°	90°									
Target	Setting1	−0.07	−0.21	−0.26	10.16	−0.91	−0.29	0.26	1.29							1.18	0.16	0.99
	Setting2	−0.07	0.09	−0.26	17.16	−1.01	−0.19	0.26	1.29							3.00	0.15	0.99
	Setting3	−0.07	0.59	−0.16	28.06	−0.91	0.11	0.26	1.19							0.71	0.09	0.99
		
*Photon*		Dose difference (%)														DIR accuracy		
		Dmean		D_1%_		D_2%_		D_5%_		D_10%_		D_20%_		D_30%_		HD (mm)	MDA (mm)	DSC
ROI	DIR setting	3D-CRT	VMAT	3D-CRT	VMAT	3D-CRT	VMAT	3D-CRT	VMAT	3D-CRT	VMAT	3D-CRT	VMAT	3D-CRT	VMAT			
OAR1	Setting1	0.06	0.03	0.16	−0.68	−0.09	−0.09	−0.11	−0.19	−0.43	−0.40	0.06	−0.13	0.04	0.05	3.01	0.04	1.00
	Setting2	−2.84	−2.37	−0.64	−2.28	−1.39	−2.29	−3.51	−3.59	−4.13	−3.60	−0.94	−2.93	−1.26	−2.55	9.03	1.27	0.90
	Setting3	−3.84	−3.37	−1.04	−2.28	−2.09	−2.49	−5.11	−4.09	−6.63	−4.30	−1.44	−3.93	−2.26	−3.65	17.21	3.03	0.78
OAR2	Setting1	−0.57	−0.55	−0.09	−0.41	−0.19	−0.24	−0.42	−0.47	−0.55	−0.42	−0.26	−0.36	−0.37	−0.45	0.52	0.08	1.00
	Setting2	−2.07	−1.75	0.31	−0.41	−0.19	−1.14	−1.22	−1.27	−2.05	−1.32	−0.96	−1.46	−1.57	−1.55	9.66	1.18	0.91
	Setting3	−2.87	−2.45	0.31	−1.51	−0.59	−1.34	−1.82	−1.97	−2.95	−2.02	−1.46	−2.26	−2.17	−2.45	16.29	3.16	0.79
OAR3	Setting1	0.01	0.04	0.26	0.10	0.12	−0.03	0.07	−0.01	−0.18	0.07	0.04	−0.01	0.08	−0.06	0.33	0.02	1.00
	Setting2	0.01	0.04	0.96	0.40	1.02	0.47	1.17	0.69	0.32	0.57	0.14	0.39	−9.92	0.14	1.64	0.31	0.98
	Setting3	−2.59	−2.76	0.56	0.20	−0.88	0.17	−1.03	−0.51	−2.98	−1.93	−1.86	−3.61	−1.22	−3.66	10.98	2.55	0.79
OAR4	Setting1	0.13	0.08	0.26	0.35	0.51	0.78	0.70	0.53	0.37	0.08	−0.08	−0.01	0.18	0.01	3.04	0.05	1.00
	Setting2	−0.97	−0.42	0.46	1.45	0.81	1.98	1.40	1.73	0.57	1.08	−0.28	−0.01	−0.52	−0.29	9.50	1.05	0.92
	Setting3	−3.57	−3.02	0.16	1.15	0.21	0.98	−0.60	−0.37	−3.33	−2.22	−2.38	−3.41	−1.92	−3.79	17.12	3.47	0.75
														
		D_90%_		D_95%_		D_98%_		D_2%_								HD (mm)	MDA (mm)	DSC
		3D-CRT	VMAT	3D-CRT	VMAT	3D-CRT	VMAT	3D-CRT	VMAT									
Target	Setting1	−0.08	−0.02	−0.12	−0.08	−0.14	−0.07	−0.06	0.01							1.18	0.16	0.99
	Setting2	−0.08	−0.02	−0.12	−0.08	−0.14	−0.07	−0.06	0.01							3.00	0.15	0.99
	Setting3	−0.08	−0.12	−0.12	−0.08	−0.14	−0.07	−0.06	0.01							0.71	0.11	0.99

**Abbreviations:** 3D-CRT, 3-dimensional conformal radiation therapy; CIRT, carbon ion radiotherapy; Dmean, mean dose; DIR, deformable image registration; DSC, dice similarity coefficient; DVH, dose volume histogram; D_x%_, minimum dose to the most irradiated x% of tissue volume; HD, Housdorff distance; MDA, mean distance to agreement; OAR, organ at risk; ROI, region of interest; and VMAT, volumetric modulated arc therapy.

**Table 2:** Difference between ground truth and warped dose of DVH parameters and DIR accuracy index values for each ROI in anatomical phantom.

Carbon PTV-based plan		Dose difference (%)														DIR accuracy		
		Dmean		D_1%_		D_2%_		D_5%_		D_10%_		D_20%_		D_30%_		HD (mm)	MDA (mm)	DSC
ROI	DIR setting	0°	90°	0°	90°	0°	90°	0°	90°	0°	90°	0°	90°	0°	90°			
OAR1	Setting1	0.03	0.04	0.16	0.12	1.30	0.95	−0.41	1.01	−0.15	−1.14	0.04	0.04	−0.04	0.00	1.65	0.06	1.00
	Setting2	0.03	0.04	0.16	0.12	1.50	1.05	−0.11	1.41	−0.15	−1.04	0.04	0.04	0.06	0.00	2.25	0.06	1.00
	Setting3	−0.47	−0.46	0.06	0.12	0.50	−0.15	−7.41	−4.09	−0.85	−4.34	−0.26	−0.06	−0.84	−0.10	6.01	0.52	0.96
OAR2	Setting1	0.08	−0.02	0.02	0.01	0.13	0.19	0.31	0.09	0.20	−0.03	0.75	−0.02	0.08	−0.02	1.78	0.08	1.00
	Setting2	0.18	0.08	0.02	0.01	0.13	0.19	0.61	0.19	0.40	0.97	1.35	0.18	0.18	0.08	2.16	0.09	1.00
	Setting3	−2.92	−1.72	0.02	0.01	−0.17	−0.11	−4.89	−5.81	−7.40	−10.53	−14.95	−4.22	−5.92	−0.82	7.33	1.64	0.91
														
		D_90%_		D_95%_		D_98%_		D_2%_								HD (mm)	MDA (mm)	DSC
		0°	90°	0°	90°	0°	90°	0°	90°									
Target	Setting1	0.00	−0.04	0.07	0.17	0.37	0.17	0.20	0.22							0.94	0.07	1.00
	Setting2	0.00	−0.04	0.07	0.17	0.37	0.27	0.20	0.22							1.53	0.09	0.99
	Setting3	0.00	−0.04	−0.03	0.07	0.27	0.07	0.30	0.22							2.30	0.13	0.99
		
*Carbon* *Robust plan*		Dose difference (%)														DIR accuracy		
		Dmean		D_1%_		D_2%_		D_5%_		D_10%_		D_20%_		D_30%_		HD (mm)	MDA (mm)	DSC
ROI	DIR setting	0°	90°	0°	90°	0°	90°	0°	90°	0°	90°	0°	90°	0°	90°			
OAR1	Setting1	0.01	−0.02	0.83	0.78	1.77	1.16	−2.31	−0.93	−0.01	−0.40	0.06	−0.05	−0.04	−0.04	1.65	0.06	1.00
	Setting2	0.01	0.08	1.03	0.78	1.77	1.26	−2.11	−0.33	−0.01	−0.30	0.06	−0.05	0.06	−0.04	2.25	0.06	1.00
	Setting3	−0.39	−0.42	0.53	0.38	−0.53	−0.64	−6.71	−6.53	−0.31	−2.50	−0.24	−0.05	−0.94	−0.04	6.01	0.52	0.96
OAR2	Setting1	0.02	0.03	0.14	0.04	0.05	0.13	−0.02	0.23	0.49	0.23	0.13	0.00	−0.02	−0.03	1.78	0.08	1.00
	Setting2	0.12	0.03	0.14	0.04	0.15	0.23	0.08	0.43	0.89	0.93	0.23	0.00	0.08	0.07	2.16	0.09	1.00
	Setting3	−2.28	−1.47	−0.06	0.04	−1.65	−1.37	−5.92	−6.47	−7.91	−11.77	−10.27	−2.30	−2.72	−0.43	7.33	1.64	0.91
														
		D_90%_		D_95%_		D_98%_		D_2%_								HD (mm)	MDA (mm)	DSC
		0°	90°	0°	90°	0°	90°	0°	90°									
Target	Setting1	0.61	0.55	0.91	0.65	1.25	0.90	0.22	0.19							0.94	0.07	1.00
	Setting2	0.61	0.65	0.91	0.65	0.85	1.00	0.22	0.19							1.53	0.09	0.99
	Setting3	0.21	0.25	0.51	0.25	0.75	0.60	0.22	0.19							2.30	0.13	0.99
		
*Photon*		Dose difference (%)														DIR accuracy		
		Dmean		D_1%_		D_2%_		D_5%_		D_10%_		D_20%_		D_30%_		HD (mm)	MDA (mm)	DSC
ROI	DIR setting	3D-CRT	VMAT	3D-CRT	VMAT	3D-CRT	VMAT	3D-CRT	VMAT	3D-CRT	VMAT	3D-CRT	VMAT	3D-CRT	VMAT			
OAR1	Setting1	−0.03	0.02	0.17	0.53	0.14	0.08	0.33	−0.45	0.14	0.01	−0.13	0.07	−0.10	−0.10	1.65	0.06	1.00
	Setting2	−0.03	0.02	0.17	0.53	0.14	0.28	0.53	−0.45	0.14	0.01	−0.13	0.07	−0.10	0.10	2.25	0.06	1.00
	Setting3	−1.23	−1.48	0.07	−0.07	−0.16	−0.72	−2.17	−1.65	−3.06	−1.89	−5.43	−1.03	−2.30	−4.80	6.01	0.52	0.96
OAR2	Setting1	0.04	0.11	−0.03	−0.02	0.03	0.07	0.05	0.06	0.41	0.06	0.27	0.31	0.15	0.37	1.78	0.08	1.00
	Setting2	0.24	0.31	−0.03	−0.02	0.03	0.07	0.25	0.16	1.01	0.36	0.77	0.81	0.25	0.87	2.16	0.09	1.00
	Setting3	−3.26	−4.39	−0.13	−0.22	−0.27	−0.53	−1.35	−2.04	−7.69	−6.24	−7.03	−6.29	−10.65	−7.23	7.33	1.64	0.91
														
		D_90%_		D_95%_		D_98%_		D_2%_								HD (mm)	MDA (mm)	DSC
		3D-CRT	VMAT	3D-CRT	VMAT	3D-CRT	VMAT	3D-CRT	VMAT									
Target	Setting1	−0.04	0.03	−0.02	0.15	−0.03	0.15	−0.01	−0.03							0.94	0.07	1.00
	Setting2	−0.04	0.03	−0.02	0.15	−0.03	0.15	−0.01	−0.03							1.53	0.09	0.99
	Setting3	−0.04	0.03	−0.12	−0.05	−0.23	−0.15	−0.01	0.07							2.30	0.13	0.99

**Abbreviations:** 3D-CRT, 3-dimensional conformal radiation therapy; CIRT, carbon ion radiotherapy; Dmean, mean dose; DIR, deformable image registration; DSC, dice similarity coefficient; DVH, dose volume histogram; D_x%_, minimum dose to the most irradiated x% of tissue volume; HD, Housdorff distance; MDA, mean distance to agreement; OAR, organ at risk; ROI, region of interest; and VMAT, volumetric modulated arc therapy.

**Figure 7:**
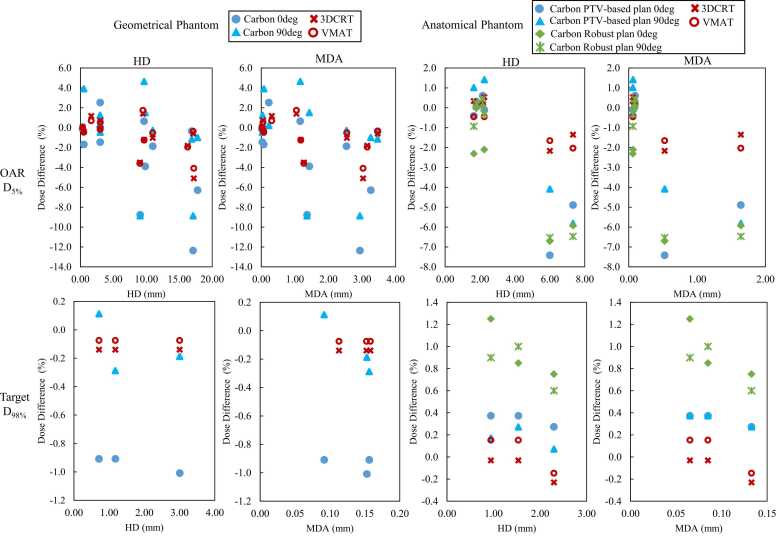
Scatterplot of the relationship between HD, MDA, and dose difference. Abbreviations: 3D-CRT, three-dimensional conformal radiotherapy; CIRT, carbon ion radiotherapy; DIR, deformable image registration; HD, Housdorff distance; MDA, mean distance to agreement; and VMAT, volumetric modulated radiotherapy.

Figure 7 shows a scatter plot of the relationship between HD and MDA and the dose difference between D_5%_ of OAR and D_98%_ of target. In both geometrical and anatomical phantoms, the CIRT plan tended to have a larger dose difference compared to the photon therapy plan, even with similar HD or MDA. For both OAR and target, the dose error was within 2% for HD and MDA <1 mm for the photon treatment plan, but for CIRT, the maximum dose difference was 7.41%, even for MDA <1 mm.

The results of the gamma analysis for the geometrical phantom and anatomical phantom are shown in Table 3, Table 4, respectively. For all criteria in both phantom images, DIR setting 1 tended to have the highest passing rate, and DIR setting 3 had the lowest pass rate. For the 3%/3 mm criterion, the passing rate >90% for most treatment plans in DIR setting1, but for the 1%/1 mm criterion, the passing rate was <80%. The gamma passing rate of criteria 1%/1 mm was significantly improved in DIR setting 1 compared to DIR setting 3 for all treatment plans.

**Table 3:** Gamma passing rate in geometrical phantom.

	DIR setting	3%/3 mm (%)	2%/2 mm (%)	1%/1 mm (%)
CIRT 0°	Setting1	95.37	90.31	76.48
	Setting2	85.85	77.57	58.31
	Setting3	65.87	55.77	37.74
CIRT 90°	Setting1	99.52	98.63	90.74
	Setting2	98.9	96.43	84.12
	Setting3	70.14	57.36	37.21
VMAT	Setting1	95.37	91.05	77.63
	Setting2	92.22	87.97	77.83
	Setting3	65.29	51.4	30.41
3D-CRT	Setting1	92.84	88.15	78.23
	Setting2	91.15	86.09	74.07
	Setting3	61.71	51.94	33.01

**Abbreviations:** 3D-CRT, 3-dimensional conformal radiation therapy; CIRT, carbon ion radiotherapy; DIR, deformable image registration; and VMAT, volumetric modulated arc therapy.

**Table 4:** Gamma passing rate in anatomical phantom.

	DIR setting	3%/3 mm (%)	2%/2 mm (%)	1%/1 mm (%)
CIRT 0° PTV-based plan	Setting1	99.87	98.72	91.54
	Setting2	99.80	98.56	90.71
	Setting3	95.57	90.38	76.01
CIRT 90° PTV-based plan	Setting1	90.05	85.53	76.46
	Setting2	81.19	75.63	62.30
	Setting3	80.86	74.63	57.40
CIRT 0° robust plan	Setting1	99.88	98.67	91.55
	Setting2	99.82	98.47	90.84
	Setting3	96.19	91.66	77.17
CIRT 90° robust plan	Setting1	89.60	84.75	75.35
	Setting2	80.17	74.48	61.70
	Setting3	79.90	73.91	56.93
VMAT	Setting1	95.29	89.48	76.80
	Setting2	84.71	74.64	58.18
	Setting3	82.57	70.26	50.82
3D-CRT	Setting1	93.82	89.42	80.68
	Setting2	83.82	77.98	65.46
	Setting3	82.21	74.92	59.92

**Abbreviations:** 3D-CRT, 3-dimensional conformal radiation therapy; CIRT, carbon ion radiotherapy; DIR, deformable image registration; PTV, planning target volume; and VMAT, volumetric modulated arc therapy.

## Discussion

In this study, we evaluated the relationship between DIR accuracy and dose deformation in CIRT. When compared with the results for photon therapy, CIRT clearly tended to have a larger dose warping error, even with similar DIR accuracy. The profiles shown in Figure 4, Figure 5 confirm the trend toward larger dose errors in the penumbral region for both photon therapy and CIRT. In CIRT, in addition to those findings, dose errors occurred in regions with steep dose gradients on the proximal and distal sides of the spread-out Bragg peak. In photon treatment planning, dose errors were consistent with the profile ground truth of the warped dose in DIR settings 2 and 1, although errors were identified in the penumbral region around the target in DIR setting 3, which had the worst DIR accuracy in this study. For CIRT, even at a warped dose for DIR setting 2, there was a wide range of errors around the target. The warped dose in CIRT had fewer errors for DIR setting 1 than for other DIR settings, but the errors occurred over a wider range than in the photon treatment plan. These results indicate that the error in dose warping may be greater for CIRT than for photon therapy, even at the same DIR accuracy. We also found that in CIRT treatment planning, the range of errors can be extensive, not only in the penumbral region but also in the proximal and distal ends. For the DVH parameters of the OAR around the target, the maximum dose error was 0.78% at its maximum in photon therapy at DIR setting 1, which is the best DIR accuracy in this study. Therefore, we considered that clinically acceptable dose warping is possible with photon therapy if the DIR accuracy is equivalent to that of DIR setting 1. In contrast, CIRT confirmed an error >3% even at DIR setting 1, especially in OARs located in the distal end and penumbral region. In addition, the warped dose at DIR setting 3, which was the least accurate in this study, had an error >10%.

There are several reports on the relationship between DIR accuracy and dose-warping accuracy in photon therapy.^36^, ^37^, ^38^, ^39^ Saleh-Sayah et al^37^ reported that deformation vector field errors ≤1 mm up to a few millimeters are acceptable in dose warping depending on the dose gradient. In comparison, the accuracy of dose warping in this study was consistent with clinically acceptable accuracy, with photon DIR accuracy of HD <1 mm and MDA < 0.1 mm and a maximum dose difference of 0.57%. Several reports have stated that the relationship between DIR accuracy and the dose warping error depends on the dose gradient.^37^, ^38^, ^39^ As shown in Figure 6, the dose gradients at the lateral penumbral and distal ends are larger in CIRT than in 3DCRT and VMAT. This may explain why the dose warping error was so large in CIRT, even with a DIR accuracy comparable to that of photon therapy. The lateral penumbra of 80% to 20% of the prescribed dose in the beam model used in this study was 7.69 mm for the carbon ion beam and 22.09 mm for the photon beam. Such differences in beam characteristics may affect the relationship between DIR and dose-warping accuracy. In the present study, there was also deformation in the superior-inferior direction, and since the dose gradients of VMAT and 3D-CRT are comparable in the superior-inferior direction, the sensitivity of dose warping in that direction to DIR accuracy was considered to be comparable. This was found to be the case in the anatomical phantom, as the dose difference for each DIR setting to the OAR2 on the superior side of the target is equivalent to VMAT and 3D-CRT (Table 2).

In this study, we used scatter plots to represent the relationship between the DIR accuracy index and the dose difference (Figure 7). In photon therapy, if HD and MDA converged to <1 mm, the dose difference of target or OARs <1%, but in CIRT, an error of >3.5% was observed even if HD was <1 mm. The results of this study revealed that, for CIRT, the dose difference for OARs around the target may be <±2% if the HD is within 0 to 5 mm and may increase by up to 7.5% every 5 mm if the HD is >5 mm. In contrast, it was found that, in photon therapy, the dose difference is <±1% within 0 to 5 mm of HD and that a maximum dose difference of 3.4% may occur every 5 mm when HD is >5 mm. Veiga et al^40^ found a clear relationship between dose gradient, DIR, and dose difference, which is consistent with our results that CIRT has a steeper dose gradient relative to photon therapy, resulting in a larger dose difference due to DIR errors. This suggests that CIRT may require more accurate DIR for dose warping accuracy comparable to photon therapy.

The results of the gamma analysis shown in Table 3, Table 4 indicate that setting 1 had the highest passing rate for both CIRT and photon treatment plans. This suggests that DIR accuracy correlates with dose-warping accuracy for both photon and CIRT. The error is generally considered to be distributed between 1%/1 mm and 2%/2 mm, because the error is generally above 90% for the 3%/3 mm criterion in DIR setting 1 but below 80% for the 1%/1 mm criterion. The gamma passing rate of 1%/1 mm of the criterion was greatly improved in DIR setting 1 compared to DIR setting 3, suggesting that the error converged within 1%/1 mm in the dose warping accuracy of DIR setting 1. It was suggested that an accuracy higher than DIR setting 1 is needed for even higher accuracy dose warping with CIRT. In the gamma analysis, there were treatment plans and tolerances with lower pass rates for 3D-CRT and VMAT than for CIRT. This is due to the fact that the dose is spread over a wide area by photon therapy and that the dose difference is large in areas far from the tumor, as shown in the dose distributions in Figure 4, Figure 5. Another factor is that the gamma analysis included not only dose but also distance as a criterion. Dose errors are more likely to occur in CIRT because of the steeper dose gradient, but errors in evaluating distance may be more likely to occur in photon therapy.

Kubota et al^24^ used DIR to evaluate the combined dose for CIRT. Their results and this study’s results are generally in agreement, revealing that a difference of <1.5 mm in MDA and >0.8 in DSC could result in a difference of approximately 2% to 3% in DVH parameters in OAR. However, these are only some of the DVH parameters, and the present study confirmed that an error of <1.5 mm MDA for some DVH parameters results in a dose warping error >4.5%. In CIRT, it is considered necessary to consider the possibility of an error of >10% even for a relatively good DIR, depending on the shape of the dose distribution, the size of the ROI to be evaluated, and the DVH parameters. For the target dose, the degree of the warped dose error did not change for either CIRT or photon therapy independent of DIR accuracy. Therefore, we considered that the same level of DIR accuracy would result in the same level of dose warping error for the target dose, regardless of the modality. However, an error >20% was identified in the CIRT 90° D_95%_ of this study, which may have been because of the location of the distal end, which is prone to intratarget errors in this treatment plan. The location of the distal end was considered important for dose warping with DIR in CIRT.

In this study, the DIR settings were the default settings in RayStation. These parameters are those used in many previous reports and are available to the general user. Therefore, the DIR accuracy realized in this study is feasible for general clinical use.^27^, ^28^, ^29^ It is known that the choice of settings and algorithms in DIR can greatly affect accuracy.^41^, ^42^ It is considered possible to achieve better DIR accuracy than in this study. Further study is important in the search for the most effective DIR in CIRT.

DIR-based dose warping accuracy results in CIRT robust plan differed from PTV-based plan for some DVH parameters (Table 2). However, for most DVH parameters, the difference between the robust plan and the PTV-based plan was minimal. The mean difference in gamma passing rates was 0.23% for all tolerances between PTV-based plan and robust plan (Table 3, Table 4). These results suggest that there is no significant difference in the sensitivity of dose warping to DIR accuracy between PTV-based plan and robust plan. In the robust plan, the target dose tended to be slightly larger than in the PTV-based plan. As shown in Figure 5, this is thought to be due to the fact that the high-dose area of the robust plan was more localized to the target. With or without robust optimization, it is considered important to evaluate where steep dose gradients occur in the dose distribution to confirm accuracy.

In actual clinical practice, it is assumed that appropriate rigid registration is performed prior to DIR. In Appendix A, we described the changes in the DIR accuracy index and the results of the gamma analysis when rigid registration was performed before DIR. The results show that proper rigid registration improves the accuracy of the DIR. In the photon therapy treatment plan, the implementation of rigid registration prior to DIR showed an improvement in the gamma pass rate of 5% to 10% at all tolerances of 3%/3 mm, 2%/2%, and 1%/1 mm. In contrast, CIRT showed an improvement in gamma pass rate at 3%/3 mm and 2%/2 mm by setting rigid registration, but the average improvement in gamma pass rate at 1%/1 mm was 3.4%, smaller than that of photon therapy. This suggests that, even when DIR accuracy is improved by implementing appropriate rigid registration in CIRT, an error of about 1%/1 mm remains. This means that CIRT requires more accurate DIR in dose warping for photon therapy.

A limitation of this study is that it was a simulation study that has not been verified by actual dose measurements. There are no reports describing the accuracy of dose warping in CIRT by dosimetry. Future validation of this study in the form of dose measurements will help establish criteria for a CIRT accuracy assessment using DIR. In this study, only 2 very limited types of images were evaluated. While we were able to evaluate quantitatively, we were unable to evaluate many variations in clinical practice. For example, head and neck cases require highly accurate registration of very complex anatomical structures. We look forward to more detailed studies with more clinical images in the future. In this study, one series of motionless images was evaluated. DIR-based dose warping is frequently utilized to evaluate cases with respiratory motion using four-dimensional CT (4D-CT).^19^, ^43^, ^44^ Since we did not validate the use of 4D-CT in this study, it will be important to quantify the DIR accuracy and dose-warping accuracy for 4D-CT in the future.

## Conclusion

We evaluated the relationship between DIR accuracy and dose-warping accuracy in CIRT. Compared with photon therapy, CIRT could result in large dose warping errors even with comparable DIR accuracy. In particular, the penumbral region and distal end of particle therapy are prone to errors, and the dose evaluation in these regions should be carefully planned and performed.

## Author Contributions

Yuya Miyasaka: Conceptualization, Writing- Original draft, Investigation. Hikaru Souda: Validation, Writing- Review and Editing. Hongbo Chai: Validation, Writing- Review and Editing. Miyu Ishizawa: Validation, Writing- Review and Editing. Hiraku Sato: Project administration, Writing- Review and Editing. Takeo Iwai: Project administration, Writing- Review and Editing.

## Declaration of Conflicts of Interest

The authors declare that they have no known competing financial interests or personal relationships that could have appeared to influence the work reported in this paper.

## Data Availability

Research data are stored in an institutional repository and will be shared upon request with the corresponding author.
