# Supplementary material for: Accuracy Evaluation of Dose Warping Using Deformable Image Registration in Carbon Ion Therapy
Source: Int J Part Ther. 2024 Dec 17;15:100639. doi: 10.1016/j.ijpt.2024.100639 (PMC11743904; doi:10.1016/j.ijpt.2024.100639)
Supplement: Supplementary file 1 — Supplementary material [file mmc1.docx]

**Appendix A.**

**Material and methods**


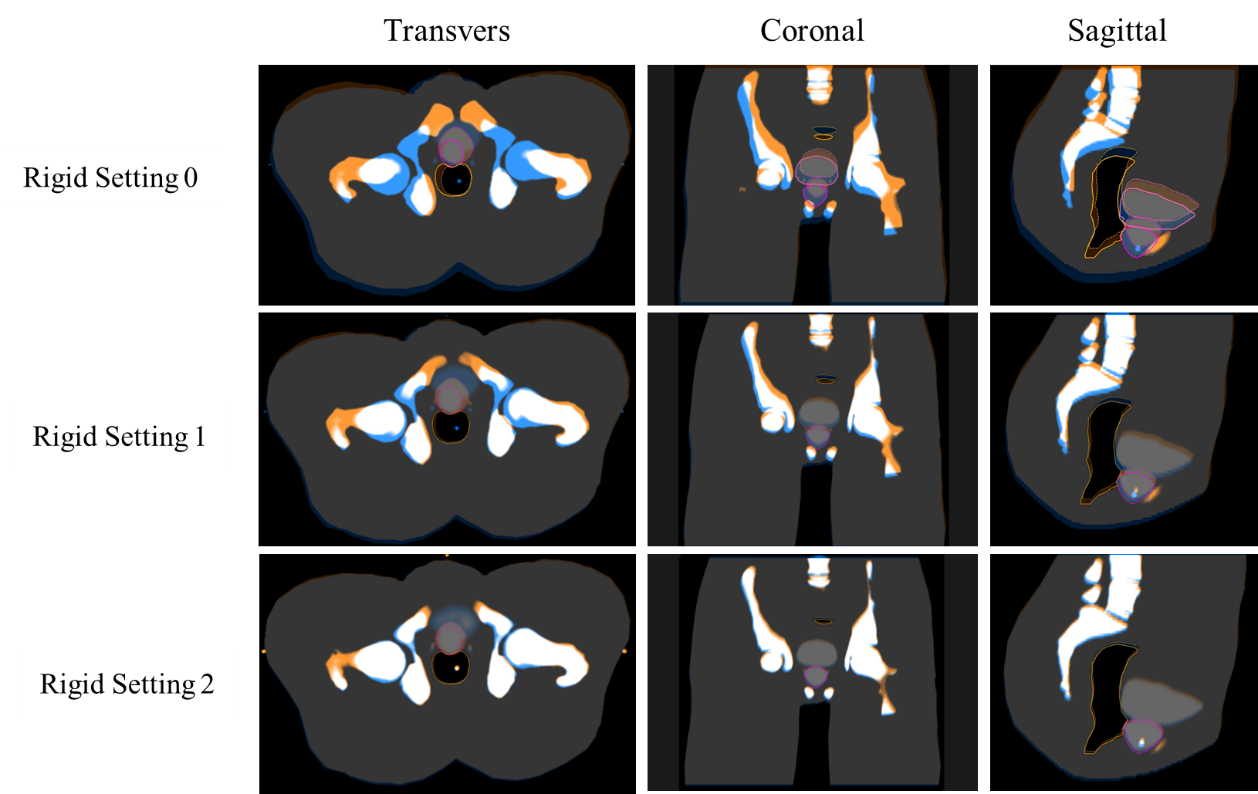
The difference between the change in DIR accuracy and the gamma pass rate was evaluated when the images were initially matched by rigid registration prior to the DIR. An anatomical phantom was used for evaluation. As in the evaluation of the main manuscript, the reference image was used as the target image and the translation image as the moving image. Rigid registration was performed in the following two settings. The displacements in the first rigid registration setting was left-right: -0.15 cm, Inferior-superior: -0.60 cm, posterior-anterior: -0.25 cm (Rigid setting 1). Rigid setting1 is a rigid registration with half the amount of shift required for the target image to perfectly match the moving image. The displacements in the second setting were left-right: -0.23 cm, inferior-superior: -0.90 cm, and posterior-anterior: -0.38 cm. (Rigid setting 2). Rigid setting 2 was a rigid registration of the amount of movement that matched the target image and the moving image even more than Rigid setting 1. The setting with no displacement, which was implemented in the study of the main manuscript, is referred to as the Rigid setting 0. The difference between the reference and translation images after each Rigid setting is shown in Figure A.1. After rigid registration of each setting, DIR settings 1, 2, and 3 were implemented as in the study in the main manuscript. The accuracy of the DIR dose warping was evaluated by comparison with ground truth doses using gamma analysis.

Figure A.1 Misalignment in each rigid setting. The blue image shows the reference image and the orange image shows the translation image.

**Results**

Table A.1. shows the results of DIR accuracy indices for DIR settings after each rigid setting. Compared to Rigid setting 0 (Table 2), there was no significant improvement in DIR accuracy in Rigid setting 1. In contrast, there was a significant improvement in DIR accuracy in Rigid setting 2. As an example, the HD at DIR setting 1 in OAR1 was 1.65 mm, 1.84 mm 0.92 mm for Rigid setting 0, setting 1, and setting 2, respectively.

Table A.2 and Table A.3 shows the results of gamma analysis of dose distributions deformed by each DIR setting after each rigid registration setting with ground truth dose. There was a significant increase in gamma pass rate in Rigid setting 2 (Table A.3) compared to Rigid setting 1 (Table A.2) at 3%/3 mm and at 2%/2 mm. For example, the increase in gamma pass rate at DIR setting 1 with a tolerance of 2%/2mm was 7.5%, 7.6%, 8.2%, and 13.0% for CIRT 90deg, CIRT 90deg Robust, 3D-CRT, and VMAT, respectively. In contrast, in photon therapy, there was a condition in which the gamma pass rate increased by more than 10% even at 1%/1mm in Rigid setting 2 compared to Rigid setting 1, but in CIRT, the increase was smaller than in photon therapy, with a maximum of 3.5% even at 1%/1 mm.

Table A.1 DIR accuracy index for each DIR setting performed after each rigid setting. (a) and (b) show the results of Rigid setting 1 and Rigid setting 2, respectively.

| (a) | Rigid setting | HD (mm) | MDA (mm) | DSC |
| --- | --- | --- | --- | --- |
| Target | Setting0 | 0.72 | 0.06 | 1.00 |
|  | setting1 | 1.14 | 0.06 | 0.99 |
|  | Setting2 | 0.69 | 0.06 | 1.00 |
| OAR1 | Setting0 | 4.73 | 0.64 | 0.95 |
|  | setting1 | 1.84 | 0.07 | 1.00 |
|  | Setting2 | 1.84 | 0.07 | 1.00 |
| OAR2 | Setting0 | 7.09 | 1.59 | 0.91 |
|  | setting1 | 2.66 | 0.09 | 1.00 |
|  | Setting2 | 2.29 | 0.09 | 1.00 |
|  |  |  |  |  |
| (b) | Rigid setting | HD (mm) | MDA (mm) | DSC |
| Target | Setting0 | 0.69 | 0.06 | 1.00 |
|  | setting1 | 0.72 | 0.06 | 1.00 |
|  | Setting2 | 0.69 | 0.06 | 1.00 |
| OAR1 | Setting0 | 3.14 | 0.41 | 0.97 |
|  | setting1 | 1.83 | 0.06 | 1.00 |
|  | Setting2 | 0.92 | 0.05 | 0.99 |
| OAR2 | Setting0 | 3.49 | 0.85 | 0.95 |
|  | setting1 | 2.29 | 0.09 | 1.00 |
|  | Setting2 | 0.94 | 0.06 | 1.00 |

Abbreviation: DIR = deformable image registration, OAR = organ at risk, HD = Housdorff distance, MDA = mean distance to agreement, DSC = dice similarity coefficient.

Table A.2 Gamma pass rate for Rigid setting 1

|  |  | DIR setting | 3%/3mm (%) | 2%2mm (%) | 1%1mm (%) |
| --- | --- | --- | --- | --- | --- |
| CIRT 0deg  PTV-based plan | | Setting1 | 99.9 | 98.6 | 91.4 |
|  |  | Setting2 | 99.9 | 98.7 | 90.1 |
|  |  | Setting3 | 95.1 | 90.2 | 75.2 |
| CIRT 90deg  PTV-based plan | | Setting1 | 88.1 | 82.4 | 70.0 |
|  |  | Setting2 | 81.3 | 72.3 | 58.8 |
|  |  | Setting3 | 79.8 | 69.7 | 53.4 |
| CIRT 0deg Robust plan | | Setting1 | 99.9 | 98.7 | 91.6 |
|  |  | Setting2 | 99.9 | 98.8 | 90.5 |
|  |  | Setting3 | 95.5 | 91.5 | 76.1 |
| CIRT 90deg Robust plan | | Setting1 | 88.7 | 82.9 | 70.3 |
|  |  | Setting2 | 82.3 | 72.3 | 58.5 |
|  |  | Setting3 | 80.9 | 70.1 | 53.0 |
| 3D-CRT | | Setting1 | 89.0 | 82.6 | 71.4 |
|  |  | Setting2 | 89.0 | 76.0 | 60.5 |
|  |  | Setting3 | 82.9 | 72.3 | 53.9 |
| VMAT | | Setting1 | 92.5 | 83.8 | 67.1 |
|  |  | Setting2 | 89.6 | 77.9 | 57.5 |
|  |  | Setting3 | 87.6 | 72.9 | 49.3 |

Abbreviation: DIR = deformable image registration, CIRT = carbon ion radiotherapy, 3D-CRT = three-dimensional conformal radiotherapy, VMAT = volumetric modulated radiotherapy, PTV = planning targe volume.

Table A.3 Gamma pass rate for Rigid setting 2

|  | DIR setting | 3%/3mm (%) | 2%2mm (%) | 1%1mm (%) |
| --- | --- | --- | --- | --- |
| CIRT 0deg  PTV-based plan | Setting1 | 100.0 | 99.0 | 88.5 |
|  | Setting2 | 100.0 | 99.1 | 87.7 |
|  | Setting3 | 99.5 | 94.6 | 77.4 |
| CIRT 90deg  PTV-based plan | Setting1 | 97.9 | 89.9 | 69.9 |
|  | Setting2 | 96.9 | 82.9 | 58.9 |
|  | Setting3 | 97.0 | 83.2 | 56.9 |
| CIRT 0deg Robust plan | Setting1 | 99.9 | 99.0 | 88.9 |
|  | Setting2 | 99.9 | 99.0 | 88.4 |
|  | Setting3 | 99.4 | 94.7 | 78.2 |
| CIRT 90deg Robust plan | Setting1 | 97.9 | 90.4 | 70.2 |
|  | Setting2 | 97.0 | 83.1 | 58.5 |
|  | Setting3 | 97.0 | 83.4 | 56.6 |
| 3D-CRT | Setting1 | 98.0 | 90.9 | 76.7 |
|  | Setting2 | 97.5 | 86.5 | 66.8 |
|  | Setting3 | 97.5 | 85.9 | 63.4 |
| VMAT | Setting1 | 100.0 | 96.8 | 79.0 |
|  | Setting2 | 100.0 | 95.3 | 70.7 |
|  | Setting3 | 100.0 | 94.4 | 66.2 |

Abbreviation: DIR = deformable image registration, CIRT = carbon ion radiotherapy, 3D-CRT = three-dimensional conformal radiotherapy, VMAT = volumetric modulated radiotherapy, PTV = planning targe volume.
